# 3D Passive Cavitation Mapping (3D-PCM) with a Large-aperture Planar Array

**DOI:** 10.21203/rs.3.rs-11022521/v1

**Published:** 2026-10-12

**Authors:** Chaorui Qiu, Daiwei Li, Haoming Huo, Arpit Mishra, Chenhang Li, Kaiping Yin, Nanchao Wang, Junqin Chen, Rui Yao, Ezra J Margolin, Michael Lipkin, Pei Zhong, Xiaoyue Ni, Junjie Yao

## Abstract

Urinary stone disease is a common urological condition with increasing incidence, particularly in developed countries. Laser lithotripsy (LL) has become a preferred minimally invasive treatment due to its high precision and low tissue damage. Recent studies suggest that cavitation plays a critical role in stone damage during LL, and three-dimensional passive cavitation mapping (3D-PCM) has emerged as a promising tool for detecting these events. However, clinical translation of 3D-PCM remains challenging due to limitations in imaging depth, field of view (FOV), and procedural compatibility. Here, we present a large-FOV dual-modality imaging system (3D-PCM and B-mode ultrasound) based on a large-aperture planar ultrasound array. Through array optimization and model-based reconstruction, our system achieves an expanded FOV of ~ 40 × 40 mm² at a clinically relevant imaging depth of ~ 110 mm, while maintaining high spatial resolution of ~ 0.6 mm laterally and ~ 0.4 mm axially. In vivo experiments in a porcine model demonstrate that the reconstructed cavitation distribution correlates well with stone damage. Our technology has the potential to provide real-time treatment feedback during LL without disrupting the standard workflow.

